# A Monogenean Gill Parasite within the Genus *Haliotrema* (Ancyrocephalidae) Infecting *Argyrops filamentosus* Fish: Morphology and Molecular Studies

**DOI:** 10.3390/ani13061010

**Published:** 2023-03-10

**Authors:** Rewaida Abdel-Gaber, Masheil Alghamdi, Saleh Al Quraishy, Esam M. Al-Shaebi, Manal F. Elkhadragy, Saeed El-Ashram, Mohamed A. Dkhil

**Affiliations:** 1 Department of Zoology, College of Sciences, King Saud University, Riyadh 12372, Saudi Arabia; 2 Department of Biology, College of Science, Princess Nourah Bint Abdulrahman University, Riyadh 11564, Saudi Arabia; 3 Faculty of Science, Kafrelsheikh University, Kafr El-Sheikh 33516, Egypt; 4 College of Life Science and Engineering, Foshan University, 18 Jiangwan Street, Foshan 528231, China; 5 Department of Zoology and Entomology, Faculty of Science, Helwan University, Cairo 11795, Egypt; 6 Applied Science Research Center, Applied Science Private University, Amman 11937, Jordan

**Keywords:** *Argyrops filamentosus*, *Haliotrema susanae*, Ancyrocephalidae, molecular identification

## Abstract

**Simple Summary:**

Fish products are a significant source of animal protein; however, they can also include various parasites, including monogeneans. All parasitic infections may spread indirectly and have an impact on human health. Many monogenean species have not yet been morphologically described. Scientists made an effort to investigate these parasites using molecular tools and the most effective genetic marker, large subunit ribosomal RNA (*28S rRNA*). The taxonomic position of the recovered species is confirmed by considering morphological and molecular data.

**Abstract:**

Due to the presence of different parasite taxa and other disease-causing agents, all fish species are extremely prone to dangers. As a result, the current study focused on some of the monogenean parasites that infect one of the economically important fish species, the soldier bream *Argyrops filamentosus*, from the Red Sea coast of Jeddah, Saudi Arabia. Following that, thirty *A. filamentosus* fish specimens were examined for monogenean parasites. The parasitic species were isolated and morphologically and molecularly studied. The presence of one monogenean species of *Haliotrema susanae* (F: Ancyrocephalidae) infecting gills was observed in 50% of the investigated fish species. The ancyrocephalid species *Haliotrema susanae* is characterized by having all generic features within the genus *Haliotrema*. It could be distinguished from other species within this genus by the male copulatory organ including a copulatory tube with no accessory piece and a haptor made up of two pairs of anchors, two bars, and seven pairs of marginal hooks. As ectoparasitic taxa of the investigated sparid fish, the current study of *Haliotrema* species constitutes the first report of this genus. A molecular phylogenetic analysis based on the partial *28S rRNA* gene region was analyzed to investigate the phylogenetic affinity of this parasite with the genus *Haliotrema* belonging to Ancyrocephalidae. This study considers the addition of a new genetic sequence for this parasite species.

## 1. Introduction

Fish are significantly high in protein and typically quite low in fat. Marine fish are the most common and widely distributed type of fish worldwide. [1]. The sparid fish fauna in the Red Sea includes 16 species from 9 different genera [2]. One of them is the king soldier bream, *Argyrops filamentosus*, which was formerly found in the western Indian Ocean, from the Red Sea, Oman, and Arabian Gulf to South Africa including Mauritius, Madagascar, and La Réunion [3,4,5]. The soldier bream is an aggressive carnivore that primarily eats benthic invertebrates and sardines [5]. The length of this fish can reach between 60 and 70 cm. Due to its great commercial worth and popularity as one of the most commonly consumed commercial fish in the region, aquaculture is a reasonable option [6].

The majority of ectoparasitic monogeneans are highly host-specific parasites that affect the skin, gills, and fins of fish [7]. Monogeneans utilize their anterior and posterior attachment apparatus, which are determined by their attachment organs, for settling, feeding, movement, and transfer from one host to another [8]. Because gills are directly involved in gas transmission, ion exchange, and maintenance of acid-base balance in the fish body, monogenean infection significantly causes heavy mortality in the population of fish. Moreover, they disrupt the site of attachment and result in localized hemorrhages. They consume the blood and cells from damaged tissue at the same time. Fish diseases that are bacterial and viral may be mechanically transmitted by monogeneans [9]. Monogeneans can be divided into two main groups, the monopisthocotyleans, which use hook-like organs on their haptors to adhere to their host, and the polyopisthocotyleans, which use clamp-like structures for attachment [9].

The Ancyrocephalidae is a family of monogeneans within a monopisthocotylean group. About 82 genera and numerous species are part of this family [10]. Since it contains roughly 210 species that infect various fish species, the genus *Haliotrema*, which was created by Johnston and Tiegs [6], is regarded as the most diversified species. *Haliotrema australe* Johnston and Tiegs [11] is the type species for *Haliotrema* that collected from the black-spotted goatfish *Upeneus signatus* (Perciformes, Mullidae). Young [12] revised the diagnostic of the genus *Haliotrema* and identified 10 additional species. Based on physical traits and host relationships, he classified the 32 *Haliotrema* species into 6 taxonomic groups. *Haliotrema* has since been assigned monogeneans with the following common traits, transforming it into a taxonomic “waste-basket group”: four anchors, fourteen marginal hooks, and two bars [13]. As a result, it has been claimed that the genus has more than 100 nominal species, all of which are parasites of teleost fish belonging to 6 orders and 33 families.

*Haliotrema* is assumed to be polyphyletic based on morphological and genetic investigations. To reconstruct a monogenean phylogenetic tree, the partial sequences of the nuclear large subunit ribosomal RNA (*28S rRNA*) gene have been employed successfully [14]. Nine *Haliotrema* species from various teleost hosts formed four clades, along with twenty-six species from other genera within the Dactylogyridae, according to a phylogenetic analysis based on the D1-D2 domain of large subunit ribosomal DNA (LSU), highlighting the clear non-monophyly of the genus [15]. Furthermore, Wu et al. [15] identified 26 species from 10 closely related genera within Ancyrocephalinae in addition to 18 extremely diversified *Haliotrema* spp. that formed numerous clades utilizing LSU and the combined LSU and partial sequence of the nuclear small subunit rDNA (SSU) datasets.

To our knowledge, Saudi Arabia had no records of the genus *Haliotrema*. To explore the monogenean species infecting the king soldier bream, *Argyrops filamentosus*, from the Red Sea in the province of Jeddah (Saudi Arabia), this study therefore combined the use of morphological and molecular approaches.

## 2. Materials and Methods

### 2.1. Fish Collection

A total of 30 fish of the solider bream fish, *Argyrops filamentosus* (F: Sparidae), were gathered from the Red Sea coast in the city of Jeddah, Saudi Arabia (21°28′ N and 39°10′ E for latitude and longitude, respectively). All fish were transported to our Laboratory of Parasitology (at the Zoology Department, College of Science, King Saud University, Riyadh, Saudi Arabia) on ice, where they were identified following the recommendations of Abu Shusha et al. [16].

### 2.2. Parasitological Examination

For parasitic infections, all fish were macro- and microscopically inspected. To eliminate mucus, fish gills were removed and submerged in normal saline and then inspected under a stereomicroscope (Nikon SMZ18, NIS ELEMENTS software) for the presence of monogenean parasites. Monogeneans were collected using fine forceps and then separated into two parts, one preserved in (4%) formalin for 2 h for the microscopic studies and the other in ethyl alcohol (96%) for molecular analysis.

### 2.3. Morphological Methods

#### 2.3.1. Light Microscopic (LM) Study

The excess fixative from the preserved specimens was rinsed away with distilled H_2_O before morphological examination [17]. Some of the fixed and flattened specimens were stained with Aceto carmine (Sigma-Aldrich, Missouri, USA) for permanent whole-mount preparation. This was followed by washing in an ascending ethyl alcohol series (70%, 80%, 90%, 95%, and 99.6%, each for 10 min), clearing in clove oil, and finally mounting in Canada balsam (Palm, 2004). A few specimens were mounted in glycerin ammonium picrate (GAP; Sigma-Aldrich, Burlington, MA, USA) to observe the organization of the terminal genitalia and the fine details of the hard components of the haptor under light microscopy (ANTI-MOULD, MICROS, Gewerbezone, Austria).

#### 2.3.2. Morphology and Morphometry

For several body parts of monogenean parasites, photomicrographs were acquired using a Leica DM 2500 microscope (NIS ELEMENTS software, ver. 3.8). According to Amine et al. [18] and Kritsky and Klimpel [19], species identification was performed based on the morphological criteria of the sclerotized attachment parts and reproductive organs. According to Al-Zubaidy [20], measurements for different body parts were acquired from digitally altered illustrations using the ImageJ 1.53e program (Wayne Rasband and contributors, National Institute of Health, USA) and expressed in micrometers (µm). The prevalence and mean intensity of the parasitic infections were established, according to the equations of Bush et al. [21].

### 2.4. Molecular Analysis

#### 2.4.1. DNA Extraction and PCR Amplification

The total genomic (g) DNA was extracted from ethanol-preserved monogenean samples using a Qiagen DNeasy tissue kit© (Hilden, Germany), following the manufacturer’s instructions. A nanodrop ND-1000 spectrophotometer was used to evaluate the concentration and purity of the genetic sample (Thermo Fischer Scientific, Inc., Wilmington, DE, USA). Polymerase chain reaction (PCR) amplification was conducted for the nuclear 28S rRNA gene. PCR amplification was performed using the primer combination of Littlewood et al. [22] and Tkach et al. [23] to amplify a partial region of the *28S rRNA* gene, LSU5 (5′-TAG GTC GAC CCG CTG AAY TTA-3′) and LSU1200R (5′-GCT ATC CTG GAG GGA AAC TTC G-3′), respectively. The thermocycling profile for PCR is performed as follows: 4 min at 94 °C, followed by 35 cycles at 92 °C (1 min), at 54 °C (1 min), 30 s at 72 °C (1 min), and 72 °C (10 min).

PCR products were examined by horizontal electrophoresis using 1.5% agarose gel (Sigma-Aldrich, MI, USA) in 1× Tris-acetate–EDTA (TAE) and post-stained with SYBR Safe DNA gel dye (Thermo Fischer Scientific, Ottawa, Canada) against the GeneRuler 100 bp Plus ready-to-use DNA ladder as a molecular weight marker (Fermentas, Lithuania); then, they were visualized with the help of a gel documentation system (Image Analyzer, Malvern, UK).

#### 2.4.2. Sequence Alignment and Molecular Phylogenetic Analysis

Sequencing was performed for PCR products using an ABI Prism Dye Terminator Cycle Sequencing Core Kit (Applied Biosystems; Thermo Fisher Scientific, Waltham, MA, USA) applied on a 310 Automated DNA Sequencer (Applied Biosystems, USA). The sequencing process was performed at Prince Naif Health Research Center (King Saud University, Riyadh, Saudi Arabia). The sequence was entered at a public sequence database, GenBank (https://www.ncbi.nlm.nih.gov/, accessed on 13 December 2022) of the National Center for Biotechnology Information (NCBI). Sequences were aligned and compared to the species that could be found in GenBank earlier. The retrieved sequence identity of the data was examined using the Basic Local Alignment Search online tool (BLAST, https://blast.ncbi.nlm.nih.gov/Blast.cgi/, accessed on 1 January 2023). Using MEGA v7.0, the phylogenetic tree was constructed. Bootstrap analysis (1000 replicates) was reported under appropriate models for nucleotide substitution with powerful branch swap filters.

## 3. Results

After the examination, 15 out of 30 (50%) of the investigated soldier bream fish, *Argyrops filamentosus*, specimens were found to have a monogenetic parasite infecting the gill region. The morphological identification criteria used to identify this parasite species led to its identification as *Haliotrema susanae* Soo [13]. Each parasitized fish had an infection intensity that did not exceed 10, with a mean of 8.28. We thoroughly investigated the morphological and molecular phylogenetic relationships of this monogenetic species.

### 3.1. Microscopic Examinations (Figure 1)

The body was elongated, fusiform with parallel lateral margins, and measured 0.440–0.688 (0.523) in length with 0.106–0.201 (0.175) as a maximum width. The tegument appeared to be smooth. The anterior region of the body was provided with three pairs of head organs, two pairs of pigmented eye spots, and glandular cells located laterally to the pharynx. The mouth was subterminal and opened ventrally. The pharynx was oval in shape and measured 0.050–0.075 (0.068) in diameter. The esophagus was very short. Ceca were bifurcated posteriorly to the pharynx and terminated blindly at the end of the body.

**Figure 1:**
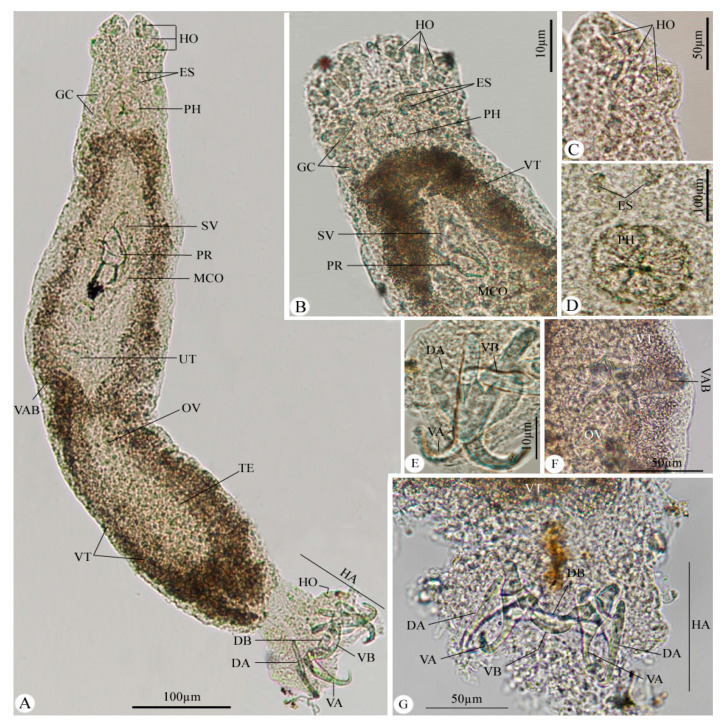
Photomicrographs of *Haliotrema susanae* infecting the soldier bream fish, *Argyrops filamentosus*. (**A**) Whole mount preparation of the parasite. (**B**–**D**) The anterior region of the body. **(E**) The ventral bar with two anchors. (**F**) The vaginal bulb and ovary. (**G**) The haptor with related structures. Note: HO, head organs; PH, pharynx; ES, eye spots; MCO, male copulatory organ; OV, ovary; SV, seminal vesicle; TE, testis; VT, vitellaria; HA, haptor; VA, ventral anchor; DA, dorsal anchor; DB, dorsal bar; VB, ventral bar; GC, glandular cells; and VAB, vaginal bulb.

The haptor set off from the body with two pairs of anchors (representing two dorsal anchors and two ventral anchors), two connecting bars (slightly bent dorsal bar and V-shaped ventral bar), and fourteen marginal hooks. The haptor was 0.068–0.113 (0.090) in length with a maximum width of 0.086–0.181 (0.123). The ventral anchor measured 0.048–0.061 (0.054), while the dorsal anchor was 0.050–0.065 (0.059). The ventral bar measured 0.037–0.056 (0.046) and the dorsal bar was 0.028–0.051 (0.044). Hooks were 0.010–0.014 (0.010) in length.

The testes were oval in shape and measured 0.091–0.110 (0.101) in length and 0.050–0.063 (0.057) in width. The vas deferens arose from the anterior portion of the testis, enlarged into a broad seminal vesicle in the mid-body, and then simply dilated, forming a saccate prostatic reservoir which was located at the anterior position to the copulatory complex. The copulatory organ consisted of a copulatory tube with no accessory piece. The male copulatory organ was measured at 0.076–0.101 (0.088) in length. 

The ovary was pyriform in shape, pre-testicular, intercaecal, and measured 0.053–0.061 (0.059) in diameter; the oviduct arose anteriorly of the ovary to form ootype and ascended anteriorly to form the uterus followed by vagina with vaginal bulb located into the mid-portion of the body. The vitellaria was densely packed throughout the trunk.

### 3.2. Remarks

This monogenean species shares the same morphological and morphometric features as *H. susanae*, which was previously isolated by Soo [13] from *Myripristis hexagona* (Holocentridae) collected from Langkawi Island, Malaysia. However, it can be differentiated from those species that infect fish in the Serranidae and Holocentridae families, such as *H. curvicirrus* Zhukov [24], in having a long copulatory organ vs. the copulatory tube of *H. susanae* having bract-like extensions from the mid-length to the initial region. Moreover, the ventral bar of the recovered species is V-shaped, similar to *H. longirectocirrus* Zhukov [24] and *H. myripristisi* Zhukov [24], but it differs from both those species in being enlarged at the ends. 

The recovered species’ dorsal bars are narrow and slightly bent in the center, similar to *H. curvicirrus*, but they are shorter than 0.044 µm in *H. susanae* and 0.033 µm in *H. curvicirrus*. The dorsal anchors of *H. susanae* have similarly shaped bases, shafts, and points to *H. papillibaculum* Zhukov [24] but differs from it by a bent and shorter copulatory tube (0.032 µm) in *H. papillibaculum* compared to the recovered species where the copulatory tube is not bent and is longer (0.088 µm).

### 3.3. Molecular Analysis

The partial *28S rRNA* sequence of the monogenetic species under study had a GC content of 50% and was 462 bp in length (A(22.29% 103) | C(20.13% 93) | G(29.87% 138) | T(27.71% 128))—deposited with the GenBank accession number OP970502.1. Nucleotide sequencing data from 31 taxa were aligned over 459 positions using the ML method to produce a phylogenetic dendrogram that represented various monogenetic species (Table 1). There was an overall mean distance between each specimen sequence of 0.175. The identification of the genus *Haliotrema* was made using a pairwise comparison with the GenBank *28S rRNA* gene dataset (Table 1). 

**Figure 2:**
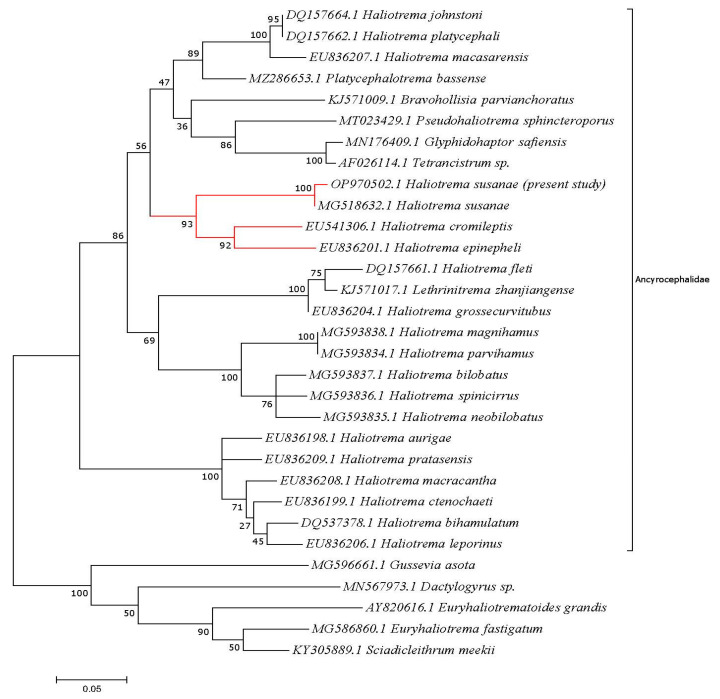
Molecular phylogenetic analysis by maximum likelihood method based on the Tamura–Nei model. The highest log likelihood (−4876.66) for this tree is shown. The percentage of trees is shown next to the branches in which the associated taxa clustered together. By applying Neighbor-Join and BioNJ algorithms, initial trees for the heuristic search were obtained automatically, to estimate a matrix of pairwise distances using the Maximum Composite Likelihood (MCL) approach, and then the topology with superior log likelihood value was selected. The tree is drawn to scale, with branch lengths measured by the number of substitutions per site.

The families Ancyrocephalidae and Dactylogyridae of the order monopisthocotylea were included in the phylogenetic study (Figure 2). The identity ranges for comparable species are 99.35–82.35% for Ancyrocephalidae and 89.58–87.82% for Dactylogyridae. The dendrogram of this study is divided into two major clades; the first is supported strongly by species from the Ancyrocephalidae, while the latter is represented by taxa from Dactylogyridae and the other genera from the former family. The recovered monogenetic species and Ancyrocephalidae species were found to be in a well-resolved separate clade on the phylogenetic dendrogram. The examined *Heliotrema* species exhibit high sequence identity. The current species was firmly placed in the same clade as the previously deposited *Haliotrema susanae* (MG518632.1), which was isolated from the gills of *Myripristis murdjan* from Malaysia. This clade had a high bootstrap value of 100.

## 4. Discussion

Monogenean parasites have received considerable scientific attention due to the significant damage they cause to fishing resources. Both marine and freshwater fish species are parasitized externally by monopisthocotyleans (Platyhelminthes, Monogenea) [25]. The ancyrocephalids that infect *A. filamentosus* (family Sparidae) are unknown as there are no studies about this group in this fish species. One Ancyrocephalidae species, *H. susanae* Soo [13], was discovered after the examination of *A. filamentosus* fish. In accordance with the previous studies [26,27,28], the infected fish only host one ancyrocephalid parasite in a unilateral gill chamber with slight protrusion of the gill cover [26,27,28].

The *Haliotrema* genus is clustered with all the ancyrocephalins sharing morphological characteristics such as the vas deferens looping around the left intestinal crus, the posterior union of the gut crura, and the dextral opening of the vagina. These characters might not be sufficient to distinguish the species at a generic level, and it should be noted that *Haliotrema* has been classified as a polyphyletic taxon by various authors [25,26,27]. *Haliotrema* was labeled a taxonomic collecting group in the previous studies of Klassen [25] and Sun et al. [28] due to the assignment of monogeneans having four anchors, fourteen marginal hooks, and two bars. The shape of the reproductive organs and haptoral structures has led to the proposal of numerous genera for the species found within *Haliotrema* [28]. The *Haliotrema* species were found worldwide in serranids and holocentrids. Only 15 specimens (50%) of the total 30 *A. filamentosus* fish evaluated for the current study exhibited a parasitic infection for the monogenean parasite in the gill region. This percentage of infection is consistent with Al-Nabati et al. [29], who identified the same occurrence level of the monogenean parasite, *Heteromicrocotyla polyorchis*, in the gill region of *Carangoides fulvoguttatus* collected from Saudi Arabia.

The current species shares all the *Haliotrema* species’ defining characteristics, making it compatible with other diplectanid species. The primary morphological characteristics of the haptoral sclerites and the reproductive organs (copulatory organ and vagina), which are generally considered for the identification of monogeneans, are used to distinguish distinct parasitic species [30,31,32]. The taxonomic identification of many parasites is currently thought to greatly benefit from the use of molecular advanced tools [33]. The partial *28S rRNA* gene sequence was employed in the current investigation as a genetic marker to confirm the phylogenetic position of monogenean species collected from the Red Sea (Saudi Arabia). To solve the phylogeny of monogeneans, Wu et al. [15], Jovelin and Justine [34], Olson and Littlewood [35], Šimková et al. [36], Plaisance et al. [37], Strona et al. [38], Yoon et al. [39], Jun [40], and Tambireddy et al. [41] reported that both the large and small subunit (LSU and SSU) of ribosomal DNA (rDNA) support the morphological identification data. The *Haliotrema* species were divided into distinct groups based on the host group and morphology in the current phylogeny, which was consistent with the results of earlier analyses by Dang et al. [42], Sun et al. [27], and Soo [13]. The polyphyletic origin of Ancyrocephalinae has been reported here; this is compatible with the previous study of Wu et al. [15].

With a high bootstrap value of 93, the monogenean parasite recovered from Saudi Arabia was grouped with *H. susanae* (infecting *Myripristis murdjan* from Malaysia), *H. epinepheli* (infecting *Epinephelus fasciatus*), and *H. cromilepti* (infecting *Cromileptis altivelis*) (the latter two fish species collected from Heron Island, Queensland, Australia). This finding agreed with the data presented by Dang et al. [36], who reported that all *Haliotrema* species clustered together based on the morphological identification criteria of anchors with long roots; the MCO lacking an accessory piece; glandular cells anterior to the MCO; and a large, thick-walled vaginal chamber. The recovered *Haliotrema* species has a maximum identity of 99.35% with *H. susanae* (MG518632.1) and clustered with it in the same clade, which Soo [13] had previously isolated from the gills of *Myripristis murdjan* (Malaysia).

## 5. Conclusions

This study provides more information about combining morphological and molecular data for the identification of *Haliotrema* species. Furthermore, the host species, *A. filamentosus*, was considered a new host, and the recovered parasite was found in a new location in Saudi Arabia. For an improved understanding of this subgroup of monogenean parasites, additional studies should be conducted using more parasite samples and genetic markers.

## Figures and Tables

**Table 1:** The monopisthocotylean taxa for the *28S rRNA* sequence were used in phylogenetic analysis.

Parasite Species	Family	Source	% Identity	GC Content
MG518632.1 *Haliotrema susanae*	Ancyrocephaliade	GenBank	99.35	49.7
EU541306.1 *Haliotrema cromileptis*	Ancyrocephaliade	GenBank	87.28	49.4
DQ537378.1 *Haliotrema bihamulatum*	Ancyrocephaliade	GenBank	88.20	50.4
EU836201.1 *Haliotrema epinepheli*	Ancyrocephaliade	GenBank	85.37	47.3
EU836207.1 *Haliotrema macasarensis*	Ancyrocephaliade	GenBank	85.46	46
DQ157664.1 *Haliotrema johnstoni*	Ancyrocephaliade	GenBank	85.43	45.2
DQ157662.1 *Haliotrema platycephali*	Ancyrocephaliade	GenBank	85.43	45
MG593838.1 *Haliotrema magnihamus*	Ancyrocephaliade	GenBank	85.89	45.7
MG593834.1 *Haliotrema parvihamus*	Ancyrocephaliade	GenBank	85.89	46.7
MG593837.1 *Haliotrema bilobatus*	Ancyrocephaliade	GenBank	85.48	47.6
MG593836.1 *Haliotrema spinicirrus*	Ancyrocephaliade	GenBank	85.24	49.2
MG593835.1 *Haliotrema neobilobatus*	Ancyrocephaliade	GenBank	84.25	48.8
EU836204.1 *Haliotrema grossecurvitubus*	Ancyrocephaliade	GenBank	83.01	46.1
DQ157661.1 *Haliotrema fleti*	Ancyrocephaliade	GenBank	82.71	46.8
EU836198.1 *Haliotrema aurigae*	Ancyrocephaliade	GenBank	83.54	49.3
EU836209.1 *Haliotrema pratasensis*	Ancyrocephaliade	GenBank	83.37	48.8
EU836208.1 *Haliotrema macracantha*	Ancyrocephaliade	GenBank	83.37	50.2
EU836206.1 *Haliotrema leporinus*	Ancyrocephaliade	GenBank	83.29	49.7
EU836199.1 *Haliotrema ctenochaeti*	Ancyrocephaliade	GenBank	83.58	50.7
MZ286653.1 *Platycephalotrema bassense*	Ancyrocephaliade	GenBank	87.67	46.5
MT023429.1 *Pseudohaliotrema sphincteroporus*	Ancyrocephaliade	GenBank	86.65	51.3
KJ571009.1 *Bravohollisia parvianchoratus*	Ancyrocephaliade	GenBank	86.08	43.4
MN176409.1 *Glyphidohaptor safiensis*	Ancyrocephaliade	GenBank	86.26	49
AF026114.1 *Tetrancistrum* sp.	Ancyrocephaliade	GenBank	86.19	48.8
KJ571017.1 *Lethrinitrema zhanjiangense*	Ancyrocephaliade	GenBank	82.35	46
MG586860.1 *Euryhaliotrema fastigatum*	Ancyrocephaliade	GenBank	84.50	47
AY820616.1 *Euryhaliotrematoides grandis*	Ancyrocephaliade	GenBank	89.58	45.4
KY305889.1 *Sciadicleithrum meekii*	Dactylogyridae	GenBank	89.58	47.4
MN567973.1 *Dactylogyrus* sp.	Dactylogyridae	GenBank	88.41	51.7
MG596661.1 *Gussevia asota*	Dactylogyridae	GenBank	87.82	48.4

## Data Availability

All the datasets generated or analyzed during this study are included in this published article.
